# Mobile-Based 3D Modeling: An In-Depth Evaluation for the Application in Indoor Scenarios

**DOI:** 10.3390/jimaging7090167

**Published:** 2021-08-29

**Authors:** Martin De Pellegrini, Lorenzo Orlandi, Daniele Sevegnani, Nicola Conci

**Affiliations:** 1 ARCODA s.r.l., 38121 Trento, Italy; lorenzo.orlandi@arcoda.it (L.O.); daniele.sevegnani@arcoda.it (D.S.); 2 Department of Information Engineering and Computer Science, University of Trento, 38123 Trento, Italy

**Keywords:** computer vision, 3D reconstruction, deep learning, indoor, digital twin, point cloud

## Abstract

Indoor environment modeling has become a relevant topic in several application fields, including augmented, virtual, and extended reality. With the digital transformation, many industries have investigated two possibilities: generating detailed models of indoor environments, allowing viewers to navigate through them; and mapping surfaces so as to insert virtual elements into real scenes. The scope of the paper is twofold. We first review the existing state-of-the-art (SoA) of learning-based methods for 3D scene reconstruction based on structure from motion (SFM) that predict depth maps and camera poses from video streams. We then present an extensive evaluation using a recent SoA network, with particular attention on the capability of generalizing on new unseen data of indoor environments. The evaluation was conducted by using the absolute relative (AbsRel) measure of the depth map prediction as the baseline metric.

## 1. Introduction

The ability of sensing 3D space using single cameras is a widely investigated topic in image processing and computer vision. Several solutions have been developed over the years to ensure the reliable reconstruction of a given environment, some adopting traditional image processing [1,2,3,4,5], and some more up-to-date learning approaches [6,7]. In fact, 3D sensing and reconstruction is a necessary building block behind a number of technologies in industry, including robotics, landslide mapping, gaming, mixed reality, archaeology, and medicine, to name a few [8,9,10]. Despite the efforts expended by the research community toward providing progressively more accurate models capable of sensing and reconstructing 3D environments, a number of challenges remain. In fact, the acquisition of 3D information can serve multiple purposes, and can be used in real-time in a multi-sensorial context, as seen in robots, and in general, autonomous systems. This often implies that the visual information is only one among the multiple inputs to a localization and navigation system. In such conditions, the potential errors emerging from inaccuracies and/or incorrect reconstruction of portions of the environment are often compensated and mitigated thanks to the presence of additional sensing devices. Vice versa, in a more restrictive context, in which multi-modal equipment is not a viable option, 3D reconstruction is performed using the visual information on its own, thereby requiring high resolution images for better feature detection, and accurate camera calibration with distortion correction in order to generate a 3D model, consisting of a sparse or dense point cloud.

In this paper, we present an in-depth evaluation of a robust state-of-the-art method for depth estimation, which is used as the core element for 3D reconstruction applications. In particular, we focus our research on indoor scenarios, in which we expect a user to collect data using an arbitrary camera, and following subjective criteria. In other words, the acquisition was not conducted by following a rigorous path to scan the environment, and thus we did not impose any constraints on the user. Such conditions are indeed very common, and cover a wide spectrum of applications, often involving workers who rely on such augmented/extended reality tools for inspection and maintenance operations.

The paper is structured as follows: in Section 2 we present some recent relevant related work. Section 3 discusses the motivation behind this work and the main contributions. In Section 4 we focus on the validation pipeline we have envisaged, describing the methodology and the metrics used. In Section 5 the results are presented and discussed. Final remarks and conclusions are presented in Section 6.

## 2. Related Work

In the following paragraphs, we report the most relevant works presented in the SoA, starting from the traditional structure from motion algorithm and surveying the most recent developments based on deep-learning. Structure from motion (SfM) [11] allows the estimation of the three-dimensional structures of objects and environments based on the motion parallax that describes the appearance changes of an object when the observer’s viewpoint changes [4]. By doing so, it is possible to infer the 3D structure of a target, and retrieve the distance from the camera to generate a 3D representation. Another basic principle of SfM is the stereo photogrammetry triangulation used to calculate the relative positions of points from stereo pairs. SfM is required to complete three main tasks. (i) Firstly, it must find correspondences between the images and measure the distances between the features extracted with respect the two image planes. Typically, SIFT [12] features are used in this phase due to their robustness against changes in scale, large variations of view point, and challenging conditions, such as different levels of illumination and partial occlusions. (ii) Second, the camera position associated with each of the images processed is computed, via bundle adjustment (BA), to calculate and optimize 3D structure, camera pose, and intrinsic calibration. (iii) Lastly, it generates a 3D dense point cloud by using the camera parameters to back project the points computed before on the 3D space, also called multiview stereo matching.

**Figure 1:**
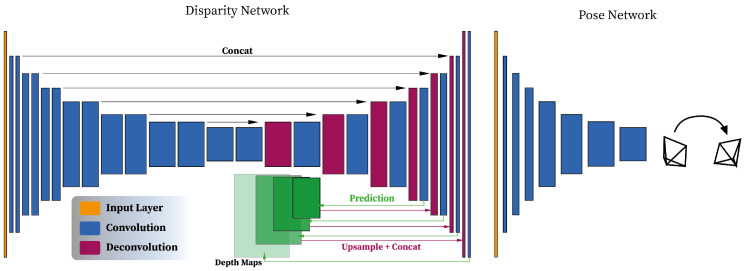
Illustration of the architecture of the Disparity estimation Network (DispNet) with the encoder–decoder layout and a pose estimation network. Additional details in terms of the size of each layer can be found in the original manuscript.

Traditional 3D reconstruction algorithms require performing heavy operations, and despite the proven effectiveness of these methods, they rely on high quality images as input. This may introduce some limitations when it comes to processing complex geometries, occlusions, and low-texture areas. Such issues have been partially tackled by replacing traditional feature and geometry-based approaches with deep learning. In particular, some stages of the traditional 3D reconstruction pipeline have been rethought following a deep learning-based formulation. Here, we present some of the methods explored, for the purpose of our research, which implement the principles of SfM using convolutional neural networks (CNNs). One of the most relevant methods exploiting neural networks for depth estimation is DispNet [13]. DispNet is used for single-view depth prediction. It uses an initial contracting stage, made of convolutional layers, followed by up-sampling to perform deconvolutions, convolutions, and computation of the loss function. Features from the contracting part are sent to the corresponding layer in the expanding portion. The network operates with a traditional encoder–decoder architecture with skip connections and multi-scale side prediction. The DispNet architecture is reported for convenience in Figure 1.

Many solutions have been developed that employ convolutional neural networks (CNNs) for the task of estimating the depth information. Some of them are used for stereo view synthesis, such as DeepStereo [14], which learns how to generate new views from single images in order to recreate a synthetic stereoscopic system where the underlying geometry is represented by quantized depth plane. Similarly, Deep3D [6] implements CNNs to convert 2D video into 3D sequences such as Anaglyph for 3D movies or side-by-side view for virtual reality (VR) applications. In this case the scene geometry is represented by probabilistic disparity maps. In addition to Deep3D, other methods can learn three-dimensional structure from a single perspective. Some of them use supervision signals, such as the method proposed by Garg et al. [15]. They proposed supervision consisting of a calibrated stereo twin for single-view depth estimation. The recent trends in depth estimation use unsupervised or self-supervised learning from video sequences. These methods work well in the task of inferring a scene geometry and ego-motion (similarly to SfM), but in addition, they show great potential for other tasks, such as segmentation, object motion mask prediction, tracking, and determining the levels of semantics (please refer to [16,17,18,19,20,21]).

**Figure 2:**
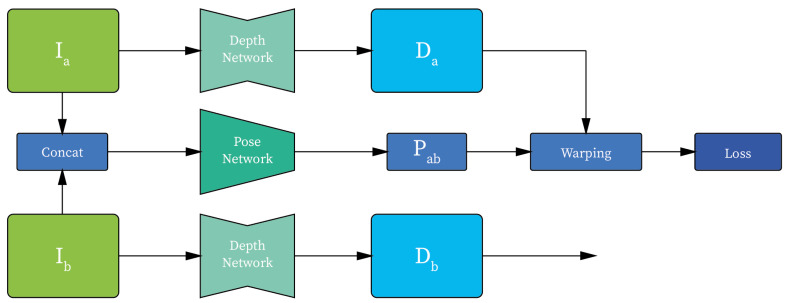
An illustration of the architecture used for the experiments, where $I_{a}$ and $I_{b}$ are the input RGB images; $D_{a}$ and $D_{b}$ are the corresponding estimated depth maps; and $P_{ab}$ is the relative camera position between $I_{a}$ and $I_{b}$.

Among the unsupervised/self-supervised methods, three important studies have been conducted by Vijayanarasimhan et al. [7], Zhou et al. [22], and Bian et al. [23]. Their approaches all implement two sub-networks: the first one focuses on single-view depth prediction, and the second one is used for camera pose estimation in support the depth network, so as to replicate a pseudo-stereo vision setting (Figure 2). These implementations mostly differ in the loss function, which is applied as a supervision signal. In terms of performances, the methods achieved state-of-the-art scores on the KITTI [24] and Cityscapes [25] datasets. Table 1 reports the methods studied.

## 3. Motivation and Contributions

Despite their proven effectiveness in street mapping contexts, the previous methods do not perform well when it comes to inferring the 3D structures of indoor environments; and training a network with indoor RGB-D datasets does not allow achieving satisfactory results, as mentioned in [26]. Indeed, DispNet aims to learn the disparity between frames, and due to the nature of hand-recorded sequences, which are typical of indoor data collection, the spatial relationship between adjacent frames might be of pure rotation, leading to a disparity equal to zero.It has been demonstrated that the estimation of the depth map is strictly related to a dominance in translation with respect to rotations in the video sequences acquisition. In fact, previous implementations have been tested on datasets such as KITTI [24], where the camera configuration and the forward motion did not give evidence to this issue. Research conducted by Bian et al. [26] has proven the existence of this limitation of DispNet, and they proposed a weak rectification algorithm to pre-process indoor datasets before training the network. The authors applied the rectification on the NUYv2 [27,28] dataset to train the network and tested the generalization capability on the *7Scene* dataset [29]. Since the generalization was evaluated on one dataset only, we provide additional benchmarks used for evaluating other RGB-D datasets and comment on the network generalization capability.

In summary, the main contributions of the paper are:

- We provide additional benchmarks for the network proposed by Bian et al. in order to allow a better understanding of the network generalization performances.
- We analyzed the network generalization capability in connection with the statistics of the scene, from which the depth was estimated. We computed the standard deviation of depth from depth ground truth to describe the amount of depth information that the network has to estimate, and then discuss how the generalization is related to this parameter.

## 4. Materials and Methods

**Figure 3:**
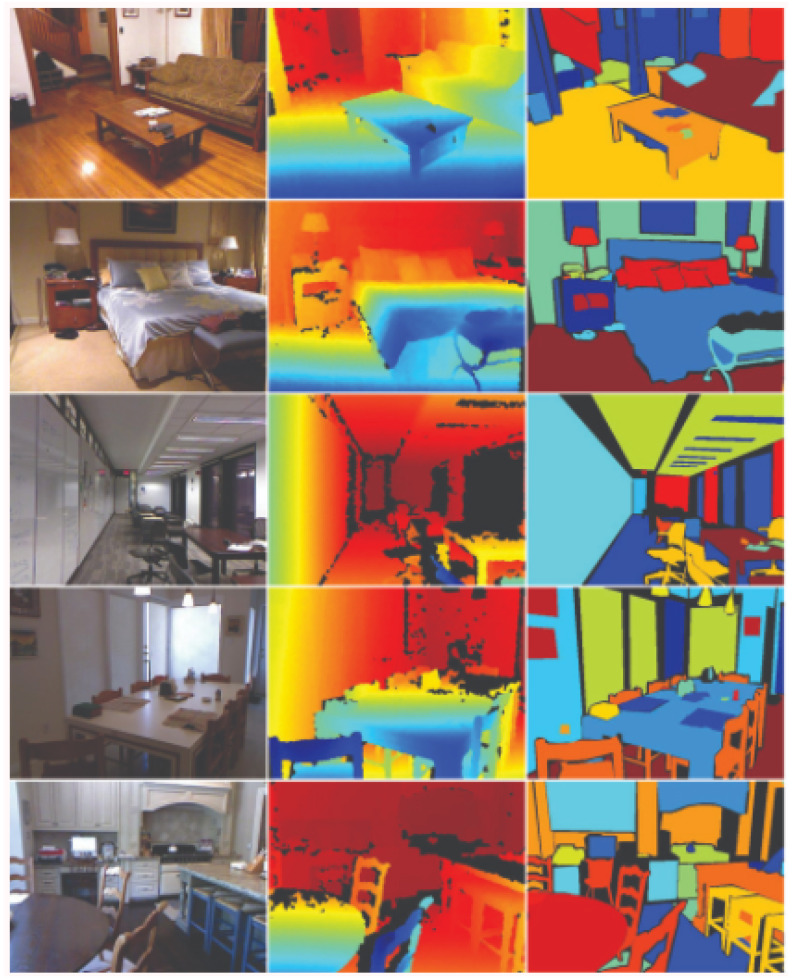
The NYU dataset [27].

As anticipated in the previous section, the results and evaluation that are presented in the following paragraphs are based on the work by Bian et al. [23,26]. Here, the network model was pre-trained on ImageNet [30] using ResNet-18 [31] in substitution of the depth and pose sub networks. Next, fine-tuning of the rectified NYUv2 (Figure 3) [27,28] dataset was applied. Differently from the other architectures, the framework was developed to overcome the scale ambiguity in [22], but it preserves the ability to test the depth and pose networks independently. We ran our first tests of depth map prediction on various RGB-D datasets of indoor environments (see Table 2), achieving results comparable to the ground truth (GT) except for a scale factor that can be calculated by normalizing the depth map with its median pixel value. The tests were conducted using the pre-trained model that is publicly available on the authors’ GitHub repository [32]. We fed the unseen datasets to the model and retrieved the predicted disparity maps. For the evaluation, we adopted the absolute relative difference, which is computed as follows:

(1) $$\frac{1}{\left|V\right|}\sum_{p\in V}\frac{|d\left(p\right)-d^{*}\left(p\right)|}{d^{*}\left(p\right)}$$

where *V* denotes the set of valid depth pixels; d(p) and $d^{*}\left(p\right)$ are the depth pixel values of the predicted depth map D and the depth ground truth $D^{*}$, respectively. As mentioned before, the predictions are different in scale with respect to the ground truth. Scaling was then applied via the scaling factor *s*, computed as follows, where med{} refers to the median value:

(2) $$s\;=\;\frac{med_{p\in V}\left\{D^{*}\right\}}{med\{D\}}$$

Note that, unlike the prediction, the ground truth exhibited some pixels equal to zero or due to reflective surfaces or distances out of the sensor range. Such non-valid pixels were discarded in the computation above.

### Datasets

The need for virtually reconstructing environments for autonomous navigation and/or extended reality applications has increased the availability of indoor RGB-D data to train more and more data-hungry networks; however, the amount of data is still limited to few common environments. In this section we present a brief overview of the datasets used in our experiments. We tested the network performance on four different datasets containing sequences from several indoor environments. In particular, for testing purposes we selected the sequences *freiburg_360* and *freiburg_pioneer* from RGB-D TUM Dataset [33], all the sequences from RGB-D 7 Scene [29], the RGB-D Scene dataset from Washington RGB-D Object Dataset [34] and the SUN RGB-D Dataset [35]. Details about the number of samples and resolution are reported in Table 2.

**Figure 4:**
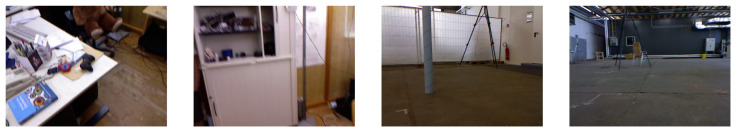
RGB-D TUM Dataset—frames taken from the two sequences.

**Figure 5:**
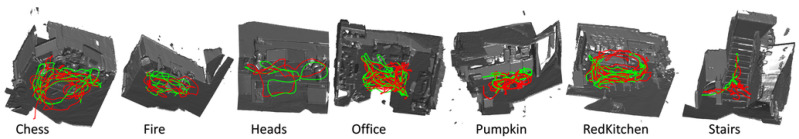
7 Scene dataset [29].

**Figure 6:**
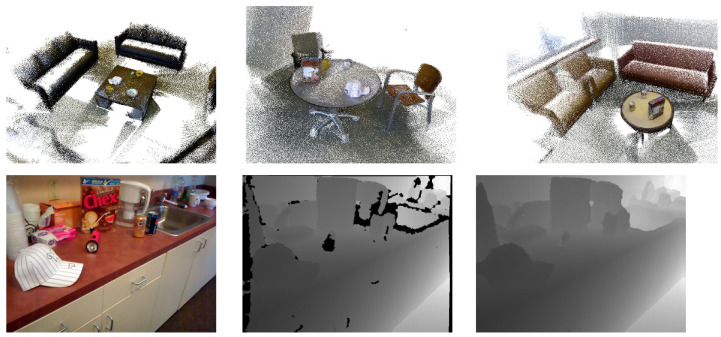
Washington RGB-D Object Dataset [34].

**Figure 7:**
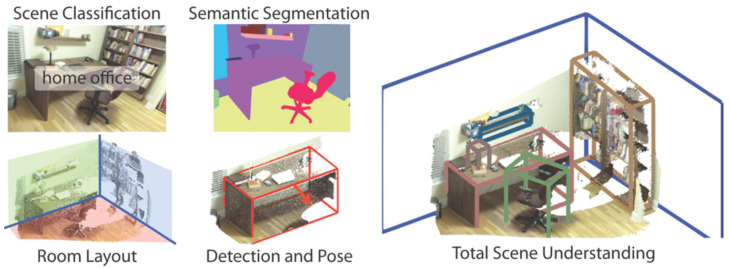
SUN RGB-D Dataset [35].

- RGB-D TUM Dataset: The sequence *freiburg1_360* contains a 360 degree acquisition in a typical office environment; the *freiburg_pioneer* sequence shows a quite open indoor environment captured by a robot with depth sensor attached on top of it (Figure 4). The dataset is provided with depth ground truth acquired by the Kinect sensor, and camera pose ground truth as rotation and translation were acquired with an external motion capture system, which is typically used for SLAM systems. For additional details we refer the reader to the dataset website [36] and to the original paper [33]. Among the available sequences we decided to choose two of them (*freiburg1_360* and *freiburg_pioneer*), since they represent distinct environments with interesting characteristics useful for testing the generalization of the network. In particular, in *freiburg_360* there are many complex geometries due to the office furniture; *freiburg_pioneer* is instead characterized by wide spaces, usually implying more homogeneous depth maps but larger depth range.
- RGB-D Microsoft Dataset: This dataset [29,37] consists of sequences of tracked RGB-D frames of various indoor environments, and it is provided with the corresponding depth ground truth (Figure 5). This dataset is the one used by the authors in [26] to test the generalization capability of the network. Accordingly, we decided to re-run the tests as well, to ensure the replicability of the paper’s results.
- Washington RGB-D Object Dataset: The dataset [34] was created with the purpose of providing structure data of real objects. Aside from the isolated objects, the dataset provides 22 annotated sequences of various indoor environment with depth ground truth. Additionally, in this case, RGB-D data were collected using Micorsoft Kinect using aligned 640 × 480 RGB and depth images (Figure 6).
- SUN RGB-D Dataset: The dataset [35] is a collection of several common indoor environments from different datasets; it contains RGB-D images from NYUv2 [28], Berkeley B3DO [38] and SUN3D [39]. The dataset has in total 10,335 RGB-D images. In order to make the experiments comparable, we have selected only the samples acquired using Kinect (Figure 7).

As reported above, in all selected datasets, the RGB-D data were acquired with Microsoft Kinect version 1. The device is equipped with an RGB camera and a structured light sensor working on the near infrared light spectrum. A known infrared pattern is projected onto the scene and the depth is computed after distortion correction. For additional information about the sensor and the related performances, please refer to the study by Wasenmüller et al. [40]. In terms of accuracy, the sensor exhibits an exponentially increasing offset going from 10 mm at 0.5 m, to 40 mm at distance of 1.8 m. Although the sensor is not as accurate as newer devices on the market, most benchmark datasets in the literature still have the Kinect depth map as ground truth.

## 5. Results

In this section we present the results we obtained in our simulations. Since the author of [26] already compared the network performances with previous state-of-the-art unsupervised methods, and in particular with [23,41], showing an improvement in terms of absolute relative error after training data rectification, we focused on enriching the benchmark by testing the network on different unseen data. We evaluated the datasets described in the previous section by feeding frame sequences into the network and computing the absolute relative difference (AbsRel) for each prediction-ground truth pair every 5 frames. The results are reported in Table 3. We notice that the network generalization performance highly depends on the images’ depth range, which has to be estimated. As an example, environments containing various structural features are more likely to result in a higher error, and frames depicting a homogeneous scenario with lower depth variations result in a lower error.

**Figure 8:**
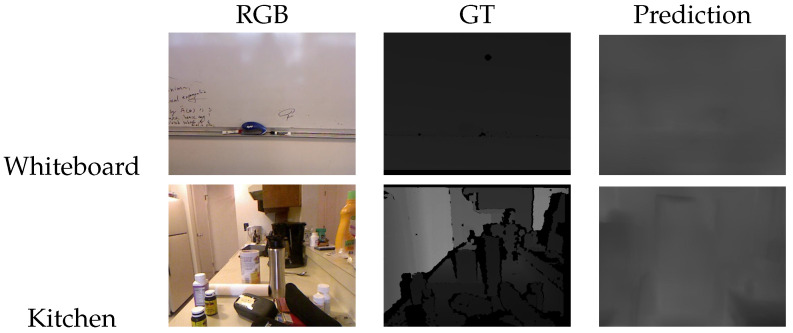
Example of depth map prediction with different standard deviation of depth.

**Figure 9:**
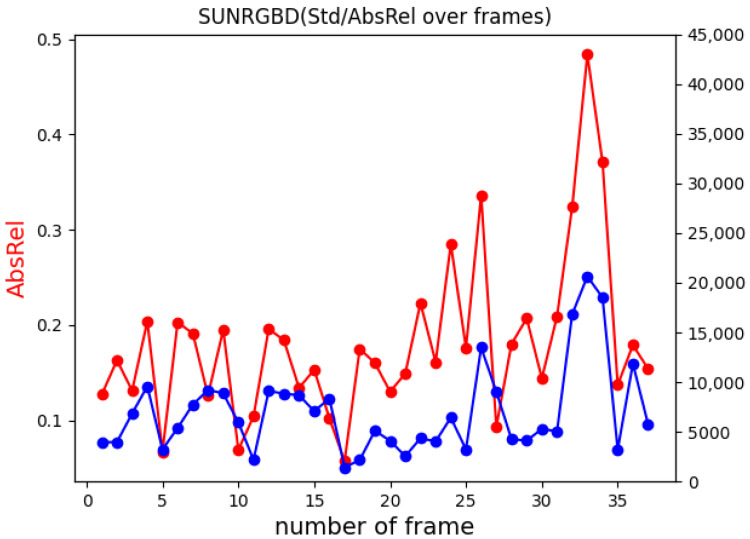
The plot shows in red the absolute relative error (AbsRel) and in blue the standard deviation of depth (Std) for the *B3DO* sequence from SUN RGB-D Dataset.

**Figure 10:**
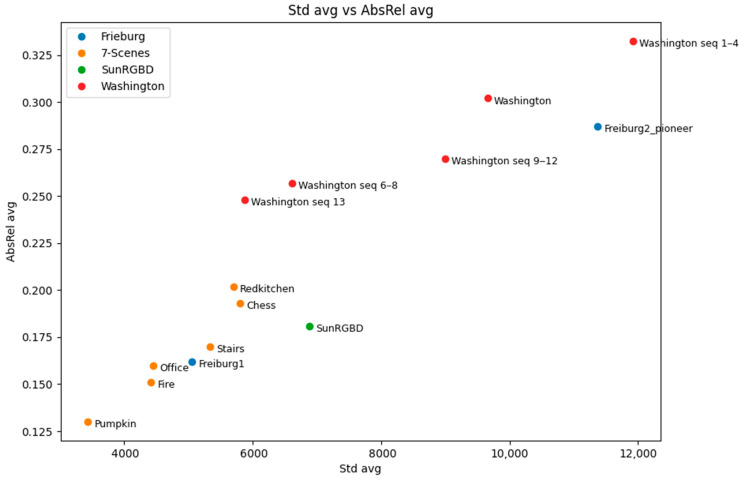
Averag standard deviation $\sigma^{2}$ vs. mean absolute relative error for every dataset.

In addition to the absolute relative error, we then analysed the standard deviation ($\sigma^{2}$) of ground truth depth images, which gives an insight into how challenging an environment is from the learning perspective. The standard deviation of depth shows great potential for understanding the overall structure of the environment; thus, it can be employed in further improvements of the network’s depth prediction. As for the AbsRel, the tests were performed by computing $\sigma^{2}$ along with the error for each frame pair for every five frames. Figure 8 shows an example of borderline situations taken from SUN RGB-D [35], where in the case of the whiteboard, the measured AbsRel is particularly low, equal to 0.05 and $\sigma^{2}=1416.48$, on the other hand, in the kitchen image the depth range is larger with $\sigma^{2}=$ 20,639.78, and the resulting absolute error is equal to 0.48. By comparing the two examples, we can see that frames with a smaller $\sigma^{2}$ consist of relatively simple tasks that the network can easily manage; at the same time they often were *false positives*. This situation was frequent because of the required normalization procedure, which was applied to the predicted depth in order to compare it with the GT. Indeed, for homogeneous surfaces that appear to be orthogonal to the optical axis, the predicted depth map resulted in an almost flat, grey, level image, leading, after the normalization, to an apparently optimal prediction, no matter whether the scale was consistent or not along the entire sequence. On the other hand, the higher the variation in the depth range, the harder is for the network to predict consistent disparity maps. This behaviour is shown in the plot reported in Figure 9, for which the tests were conducted on the *B3DO* sequence from SUN RGB-D. Unlike the other sequences, *B3DO* is composed by random frames from different environment; thus, it was a good challenge for the generalization capabilities of the network. As next step we performed the same test on the remaining data (Table 2) to find the contexts in which the network works well and in which ones it is harder for the network to predict the disparity. Figure 10 presents the absolute relative error for each considered sequence in relation to the standard deviation of depth both computed as the mean over the entire sequence. It is arguable from the plot that the absolute relative error is directly proportional to the amount of depth information (given by the standard deviation) that the network has to estimate. More precisely, it is noticeable that for datasets such as 7Scene, SUN RGB-D, and the sequence *freiburg_360*, where the space is limited and so the overall standard deviation of depth is limited, the network tended to remain consistent and more accurate in its predictions, resulting in lower absolute error. On the other hand, the prediction accuracy decreased when it came to processing wider and more complex environments: the ones belonging to the Washington dataset and the sequence *freiburg_pioneer*, and this was due to the higher variations in the environmental depth, as can be seen in Figure 10.

## 6. Conclusions

The goal of our paper was to test the generalization performance of the architecture proposed in [23], providing additional benchmark evaluations. The evaluations have been conducted using the absolute relative error as the standard metric. In addition, we aimed at providing the reader with some hints for interpreting the reasons behind some of the results we achieved, so as to draw more detailed conclusions. We noticed that the network’s ability to estimate the structure of an indoor environment is related to the amount of information that has to be learnt, as can be seen in the plots reported above. In particular, the Washington dataset provided the worst results, and this was mostly due to the larger standard deviation of the depth range. We understand that this parameter can be considered as a valuable parameter to describe the network’s generalization capability for various environments. According to our experience, we believe that employing the standard deviation of depth as a weighting parameter in the learning stage is useful, to better stimulate the network’s prediction of consistent disparity maps from large and more complex indoor environments.

## 7. Future Works

We tried to extend the evaluation of DispNet in a diversified set of scenarios, with the purpose of testing the depth extraction accuracy in monocular videos, using (SoA) CNN. It is needless to say how such an approach would be revolutionary when deployed in real and unconstrained scenarios, and could prove to be valuable for the companies engaged in the collection of digital twins, and for the ones involved in mixed and augmented reality developments. Our aims and recommendations for future studies include:

- The adoption of other SoA architectures for richer comparisons;
- The adoption of a novel metric that considers the standard deviation of depth for performance evaluations and the training stage;
- The extension of the study to additional datasets, for which the ground truth has been collected with more up-to-date and accurate depth sensors.

## Figures and Tables

**Table 1:** Methods from the literature for depth estimation from video sequences. In the column Note, symbols (O) and (R) refer to original and rectified training data.

Ref.	Method	Indoor	Dataset	Note
[7]	SfM Net	✗	KITTI [24] & Cityscapes [25]	O
[22]	SfM Learner	✗	KITTI [24] & Cityscapes [25]	O
[23]	SC-SfM Learner	✗	KITTI [24] & Cityscapes [25]	O
[26]	Indoor SC-SfM Learner	✓	NYUv2 [27,28]	R

**Table 2:** Details of the three datasets used in the testing phase, the italic format indicates a specific sequence from the dataset specified between brackets.

Name	#Images	Img. Size	Ref.
7Scene	29,000	640 × 480	[29]
*freiburg_360* (TUM RGB-D)	756	640 × 480	[33]
*freiburg_pioneer* (TUM RGB-D)	1225	640 × 480	[33]
Washington	11,440	640 × 480	[34]
SUN	10,335	640 × 480	[35]

**Table 3:** Single-view depth estimation results on selected datasets.

Scenes	AbsRel	StdDev ($\sigma^{2}$)
*Chess* (7Scene) [29]	0.19	5800.00
*Fire* (7Scene) [29]	0.15	4418.00
*Office* (7Scene) [29]	0.16	4438.00
*Pumpkin* (7Scene) [29]	0.13	3435.00
*RedKitchen* (7Scene) [29]	0.20	5700.00
*Stairs* (7Scene) [29]	0.17	5341.00
*freiburg_360* (TUM RGB-D) [33]	0.16	5056.86
*freiburg_pioneer* (TUM RGB-D) [33]	0.28	11,370.31
Washington [34]	0.3	9656.00
*B3DO* (SUN RGB-D) [35]	0.18	6886.21

## Data Availability

All data supporting reported results are public.

## Abbreviations

The following abbreviations are used in this manuscript:

SoA	State-of-the-art
SfM	Structure from motion
SIFT	Scale invariant feature transform
BA	Bundle adjustment
CNN	Convolutional neural network
DispNet	Disparity network
RGB	Red, green, blue
RGB-D	Red, green, blue and depth
GT	Ground truth
AbsRel	Absolute relative error
StdDev	Standard deviation
